# Molecular characterization of a cathepsin F-like protease in *Trichinella spiralis*

**DOI:** 10.1186/s13071-015-1270-y

**Published:** 2015-12-21

**Authors:** Zi-gang Qu, Xue-ting Ma, Wen-hui Li, Nian-zhang Zhang, Long Yue, Jian-min Cui, Jian-ping Cai, Wan-zhong Jia, Bao-quan Fu

**Affiliations:** State Key Laboratory of Veterinary Etiological Biology, Key Laboratory of Veterinary Public Health of the Ministry of Agriculture, Key Laboratory of Veterinary Parasitology of Gansu Province, Lanzhou Veterinary Research Institute, Chinese Academy of Agricultural Sciences, Lanzhou, 730046 P. R. China; Jiangsu Co-innovation Center for Prevention and Control of Important Animal Infectious Disease, Yangzhou, 225009 P. R. China

**Keywords:** *Trichinella spiralis*, Cysteine protease, Cathepsin F, Enzyme activity

## Abstract

**Background:**

Trichinellosis is a re-emerging infectious disease, caused by *Trichinella* spp. Cathepsin F belongs to cysteine protease that is a major virulence factor for parasitic helminths, and it may be a potential anti-helminth drug target and vaccine candidate. The aim of this study was to clone, express and identify a cathepsin F-like protease in *Trichinella spiralis* and to investigate its biochemical characteristics.

**Methods:**

The full-length cDNA encoding a putative cathepsin F-like protease in *T. spiralis*, TsCF1, was cloned and its biochemical characterization and expression profile were analyzed. Transcription of TsCF1 at different developmental stages of *T. spiralis* was observed by RT-PCR. The recombinant TsCF1 protein was expressed by prokaryotic expression system and recombinant TsCF1 (rTsCF1) was analyzed by western blotting. And expression of TsCF1 at muscle larvae stage was performed by immunofluorescent technique. Molecular modeling of TsCF1 and its binding mode with E-64 and K11777 were analyzed. Enzyme activity and inhibitory test with E-64 as inhibitor were investigated by using Z-Phe-Arg-AMC as specific substrate.

**Results:**

Sequence analysis revealed that TsCF1 ORF encodes a protein of 366 aa with a theoretical molecular weight of 41.9 kDa and an isoelectric point of 7.46. The cysteine protease conserved active site of Cys173, His309 and Asn333 were identified and cathepsin F specific motif ERFNAQ like KLFNAQ sequence was revealed in the propeptide of TsCF1. Sequence alignment analysis revealed a higher than 40 % identity with other cathepsin F from parasitic helminth and phylogenetic analysis indicated TsCF1 located at the junction of nematode and trematode. RT-PCR revealed the gene was expressed in muscle larvae, newborn larvae and adult stages. SDS-PAGE revealed the recombinant protein was expressed with the molecular weight of 45 kDa. The purified rTsCF1 was used to immunize rabbit and the immune serum could recognize a band of about 46 kDa in soluble protein of adult, muscle larvae and ES product of muscle larvae. Immunolocalization analysis showed that TsCF1 located on the cuticle and stichosome of the muscle larvae. After renaturation rTsCF1 demonstrated substantial enzyme activity to Z-Phe-Arg-AMC substrate with the optimal pH 5.5 and this activity could be inhibited by cysteine protease inhibitor E-64. Further analysis showed the kinetic parameters of rTsCF1 to be Km = 0.5091 μM and Vmax = 6.12 RFU/s μM at pH 5.5, and the IC_50_ value of E64 was 135.50 ± 16.90 nM.

**Conclusion:**

TsCF1 was expressed in all stages of *T. spiralis* and localized in the cuticle and stichosome. TsCF1 might play a role in the life cycle of *T. spiralis* and could be used as a potential vaccine candidate and drug target against *T. spiralis* infection*.*

## Background

*Trichinella spiralis* is a zoonotic nematode featured by causing chronic, debilitating infections with considerable morbidity and mortality. The life-cycle stages of *T. spiralis* constitute by adults worm (Ad), muscle larvae (ML) and newborn larvae (NBL). After ingestion of meat contaminated with *T. spiralis* ML, the parasite can invade into intestinal epithelium, and develop Ad via four molts within 3 to 4 days. NBL are produced at 4 to 10 days post-infection (dpi) according to the host species. NBL then migrate through the lymphatic and blood vessels and invade into host striated muscle cells and further develop into the muscle larvae, which can be infective to new host. The processes of migration and invasion are involved in a complex host-parasite interaction, and this peculiar stage is maintained until being ingested by a new host, thereafter the next generation begins [1].

Parasite proteases may be involved in a wide variety of adaptive functions and play key roles as virulence factors in many parasite infections. Cysteine proteases have been well characterized in numerous helminths and could be key molecules in host-parasite interactions [2–6]. They participate in excystation, molting, tissue penetration, catabolism of host proteins for nutrition, and immune evasion. Some of them have been revealed to be potential drug targets for antihelminth agents, chemotherapy or immunoprophylaxis, vaccine antigen candidate as well as immunodiagnosis [7–9]. One of the most studied parasite proteases is the cysteine protease from clan CA, family C1, which contains cathepsin B, C, F, H, K, L, O, S, V, W and X. In parasitic helminths, cysteine proteases in this family are especially well studied and several cathepsin Fs have been characterized in parasitic nematode and trematode [10–13].

Although several proteases have been reported in *T. spiralis,* most of them are serine protease [14–17] and few cysteine proteases have been identified in *T. spiralis* [18, 19]. In this study, a cathepsin F-like protease gene in *T. spiralis* (TsCF1) was cloned and identified. The expression, immunolocalization of TsCF1 and biochemical properties of recombinant TsCF1 protein were also characterized*.*

## Methods

### Ethics statement

Animals were treated in strict accordance with the Animal Ethics Procedures and Guidelines of the People’s Republic of China. All animal procedures were approved by the Animal Ethics Committee of the Lanzhou Veterinary Research Institute, Chinese Academy of Agricultural Sciences.

### Parasites and antigen

*T. spiralis* (ISS534) ML were isolated from infected mice at 35 dpi by artificial digestion with pepsin-HCl. The protocols of isolation of Ad from infected rats intestines and NBL from female adults were followed as previous study [14]. Soluble antigens of *T. spiralis* and excretory/secretory antigen were prepared according to the method reported [20].

### Cloning of TsCF1 gene

Key words “*Trichinella spiralis* AND cathepsin F” were input for Gene database of GenBank search. Three cathepsin F-like genes (Tsp_02382, Tsp_04256 and Tsp_02383) were acquired and named TsCF1, TsCF2 and TsCF3 respectively. Total RNA was extracted from the ML at 35 dpi, Ad and NBL of *T. spiralis* using Trizol (Invitrogen). Primer PR1 (5′-GCGAATTCTGCAGGATCCAACTTTTTTTTTTTTTTTTTT-3′) was used as the reverse transcription primer. Primer 1 (5′-CATCATTATGGTTTCCGTA-3′) and primer 2 (5′-GCGAATTCTGCAGGATCCAAC-3′) were used to amplify TsCF1 gene and study the transcription at different developmental stages. The PCR products were purified, ligated into pMD18-T vector (Takara Bio, Japan) and sequenced. Analysis of the deduced amino acid sequence was conducted with DNASTAR (DNASTAR, Madison, WI, USA) and http://www.expasy.org/tools/.

### Molecular modeling of TsCF1

Homology modeling of the mature domain of TsCF1 was performed with MODELLER v9.5 [21]. In terms of the improvement in model quality, an advanced modeling approach that is based on multiple templates of MODELLER was chosen. Thus, 1M6D (Crystal structure of human cathepsin F), 2P86 (cathepsin L protease from *T. brucei rhodesiense*) and 3BCN (Crystal structure of a papain-like cysteine protease) were selected as templates for homology modeling. Alignment between the target sequence and templates were performed using SALIGN [22] in MODELLER. New models of target sequence were built based on the multiple templates alignment. The structure of TsCF1 generated by MODELLER was improved by energy minimization using the Discovery Studio 2.5 (Accelrys Inc., San Diego, CA, USA). After energy minimization, structures were submitted to the website of PDBsum Server (http://www.ebi.ac.uk/thornton-srv/databases/cgi-bin/pdbsum/GetPage.pl?pdbcode=index.html) (PROCHECK program) and the Structural Analysis and Verification Server (http://services.mbi.ucla.edu/SAVES/) (ERRAT program), and generated a full set of structural analysis for models.

Ligands E64 and K11777 were docked into the activity pocket of the receptor TsCF1 using AUTODOCK 4.0 [23]. Lamarckian genetic algorithm (LGA) was used to search for the optimized conformation. The maximum number of generations and energy evaluations were set to 2.7 × 10^5^ and 2.5 × 10^6^, respectively. The conformations with lowest binding free energy of all docking results were selected and hypothesized to be a representative binding mode of ligand and receptors. Energy minimizations were performed to produce a series of more meaningful complex conformations for analysis. The complex structures for analysis were minimized with the steepest descent and conjugate gradient by Discovery Studio 2.5.

### Phylogenetic analysis of TsCF1

To explore the phylogenetic relationships involving *T. spiralis* TsCFs and cathepsin Fs of other helminths, a rooted Bayesian (BI) phylogenetic tree was constructed by MrBayes v3.2.2 (https://www.phylo.org/) under the best-fit model WAG + I + G [24, 25]. The best-fit model (WAG + I + G) for amino acid substitution was selected using ProtTest v2.4 with discrete gamma distribution in four categories [26]. For each model, the MCMC was run for 2 million steps and sampled every 500 steps. The first 500,000 steps of each run are discarded as burn-in. The tree was visualized using a FigTree program v1.4.0 (http://tree.bio.ed.ac.uk/software/figtree/).

### Expression, purification and refolding of recombinant TsCF1

The TsCF1 prodomain and the entire mature domain were amplified using the specific primers carrying *Xho* I and *Bam* HI restriction enzyme sites: PF2 (5′-CGCGGATCCTTGCCAATGAAGCAAAAGAGA-3′) and PR2 (5′- CGCCTCGAGTTAATCAATCACAACTGA-3′) with pMD18T-TsCF1 plasmid as the template. Then the amplified PCR product was ligated into pET-30a vector (Novagen, USA) which was digested by *Xho* I and *Bam* HI and the recombinant plasmid was transformed into *E. coli* BL21 (DE3) cells (Transgen). The expression of the recombinant TsCF1 protein (rTsCF1) was induced by adding isopropyl-1-thio-β-d-galactopyranoside (IPTG) to a final concentration of 1 mM and analyzed on Coomassie-stained SDS polyacrylamide gel electrophoresis (SDS-PAGE) gels. After SDS-PAGE, protein bands were excised from SDS-PAGE zymography gels and stored in ultrapure water. MALDI -TOF/TOF-MS/MS was performed by 4800 Plus MALDI TOF/TOF TM Analyzer (Applied Biosystems, Foster City, USA) in Shanghai Applied Protein Technology Co., Ltd (Shanghai). The MS data were used to search against the non-redundant protein database (nr database, NCBI).

To purify rTsCF1, the cells were harvested at 4 h after incubation with IPTG at 37 °C, and then the pellet of cells from 2,000 ml of culture were suspended in 8 M urea lysis buffer. The rTsCF1 was purified by nickel-nitrilotriacetic acid (Ni-NTA) chromatography (Merck, Germany), and the purity was analyzed by SDS-PAGE. Refolding of the purified rTsCF1 was performed as follows: Ni-NTA affinity purified rTsCF1 (10 mg) was slowly added into 1 L of 100 mM Tris–HCl (pH 8.0) containing 1 mM EDTA, 250 mM l-arginine, 5 mM reduced glutathione, 1 mM oxidised glutathione and gently stirred at 4 °C for 20 h as described previously [12].

### Production of rabbit polyclonal antibody and Western blot analysis

Two New Zealand rabbits were used to produce the polyclonal antibodies against rTsCF1. Rabbits were primarily immunized subcutaneously at two locations with 1 mg rTsCF1 mixed with Complete Freund′ Adjuvant. Booster immunizations were performed at 3 week intervals with 1 mg rTsCF1 emulsion of incomplete Freund′ Adjuvant. The blood samples were collected prior to each immunization, and 7 days after the last immunization, the antibody titers were checked by ELISA.

The extracts from *T. spiralis* Ad, ML and E/S antigens as well as rTsCF1 were separated on 12.5 % SDS-PAGE before being electrophoretically transferred onto Hybond C extra membranes (Amersham, USA). The membrane were then blocked and incubated with 1:200 diluted anti-rTsCF1 rabbit sera. After washing, the membranes were incubated with goat anti-rabbit immunoglobin G (IgG) alkaline phosphatase (AP) conjugate (1:4,000). Finally, the bands were detected using NBT/BCIP (Promega, USA).

### Immunolocalization

ML of *T. spiralis* were recovered from infected mice by the acid-pepsin digestion of striated muscles at 35 dpi. The parasites were fixed in cold acetone (−20 °C) for 10 min and then centrifuged for 5 min at 1000 *g*, followed by washing three times in PBS. After treated with 0.1 % Triton X-100 for 10 min at room temperature, the parasites were blocked with 5 % normal goat serum in PBS and then incubated in a moist chamber at 37 °C for 1 h with a 1: 10 dilution of immune and normal rabbit sera. After washing three times in PBS, the larvae were incubated with a 1: 20 dilution of FITC-labeled goat anti-rabbit IgG (Sigma-Aldrich, USA) and 0.01 % Evan’s blue for 1 h, washed five times in PBS, then transformed to slides and added 90 % Buffered Glycerol then observed under a fluorescent microscope (Leica, Germany).

### Enzyme activity assay and inhibitory test

Enzyme activity was assayed fluorometrically as the hydrolysis of Z-Phe-Arg-AMC (Peptide Institute, Osaka, Japan). Briefly, 0.2 μg of enzyme was added to 190 μl of sodium acetate buffer (pH 5.5) containing 2 μM Z-Phe-Arg-AMC and 10 mM DTT, and the release of fluorescence (excitation 355 nm, emission 460 nm) over 20 min at room temperature was assessed with a SpectraMax M5 (Molecular Device Corporation, Florida, USA).

For evaluation of the inhibitory kinetic features of rTsCF1, a known inhibitor E64, was selected for assay [27]. The 50 % inhibitory concentration (IC_50_) values of E64 of rTsCF1 was measured in a fluorescence endpoint assay using Z-Phe-Arg-AMC as a substrate [28]. The assay was carried out in black 96-well plates at 37 °C in a 50 mM sodium acetate buffer at pH 5.5 containing 10 mM DTT, 5 mM EDTA, and 2 μM Z-Phe-Arg-AMC. Prior to the addition of substrate, different concentration of the inhibitor ranging from 0.1 to 200 nM were preincubated for 30 min with the enzyme to allow the establishment of the enzyme-inhibitor complex. The reaction was started by the addition of the substrate and stopped after a 20 min reaction at 37 °C according to the previously described method. All values used were mean values from three independent assays to produce statistically significant results.

## Results

### Cloning and identification of the cDNA encoding TsCF1

**Fig. 1:**
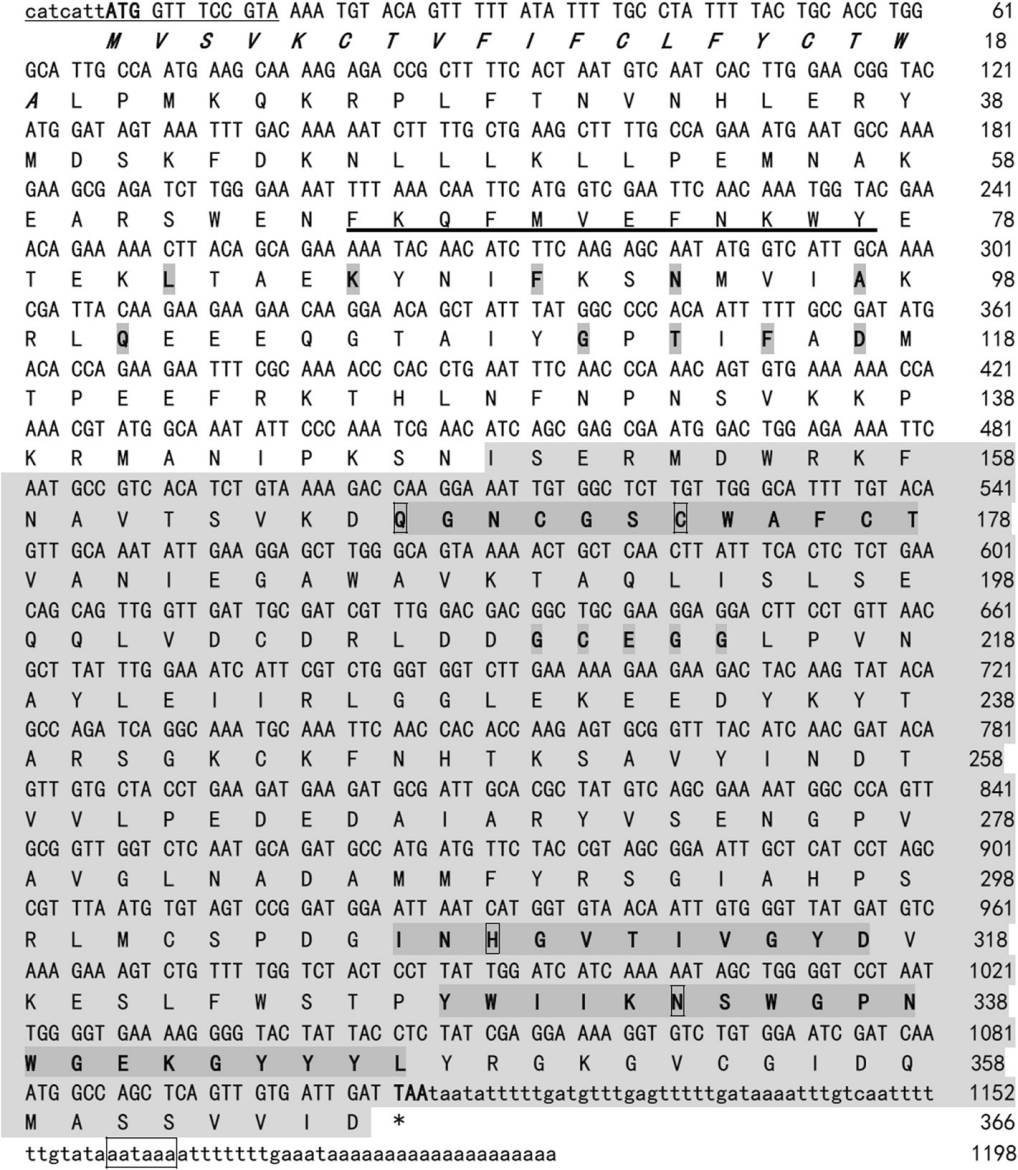
The nucleotide and deduced amino acid sequence of TsCF1 cDNA. The mature protease domain is shown *lightly shaded*; the conserved KLFNAQ, GNFN and GCNGG motifs are shown *lightly shaded and* in *bold*; and conserved papain family active site amino acids are indicated in the *square frame* of the sequence

**Fig. 2:**
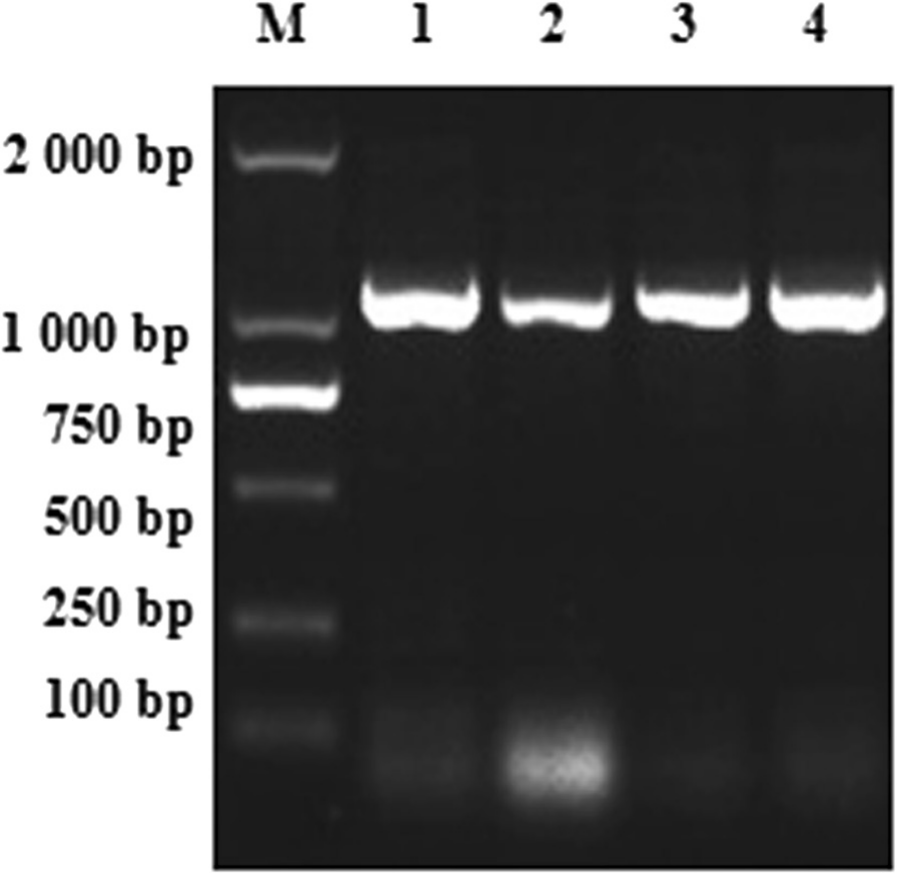
RT-PCR results of TsCF1 gene at different stages of *T. spiralis.* M: DL2000 DNA Marker; 1: muscle larvae; 2: newborn larvae; 3: 3 day old adult; 4: 5 day old adult

The TsCF1 cDNA fragment of 1198 bp was cloned and sequence analysis revealed an open reading frame of 1101 bp (Fig. 1). The deduced protein consisted of 366 amino acids with a theoretical molecular weight of 41.9 kDa and an isoelectric point of 7.46. Analysis of secondary structure revealed 31.4 and 15.3 % of α-helix and β-strands, respectively. A signal peptide sequence between amino acid 1 and 19 and 3 potential N-glycosylation sites were predicted. TsCF1 was predicted to be a typical cathepsin-F-like cysteine peptidase with a large prodomain (amino acid 20–148) and a mature domain (amino acid 149–366). The conserved motifs in the cathepsin F cysteine protease family, ERFNAQ like LKFNAQ sequence, GNFN and GCNGG, were found in the pro-and mature domains. The potential lysosomal targeting signal sequence FKQFMVEFNKWY was also found in the prodomain. Six residues Cys (at position 302, 354, 170, 211, 204 and 244) constituted disulfide bonds which are highly conserved among the papain-like cysteine proteases. The predicted active pocket containing the core residues Gln167, Cys173, His309 in TsCF1 was responsible for the catalytic activity of enzyme. RT-PCR indicated that TsCF1 was expressed in ML, Ad and NBL stages of *T. spiralis* (Fig. 2).

### Molecular modeling of TsCF1 protein

The final model was validated and assessed using the Structural Analysis and Verification Server to evaluate the quality of models. The structural analysis of homology model with Procheck showed 77.5 % of residues to be located in the most favorable geometric parts of the Ramachandran plot, 18.5 % in the additionally allowed parts, and 3.5 % in generously allowed regions, which indicated that the backbone dihedral angles φ and ψ in the model is reasonably accurate. TsCF1 was modeled with overall quality factors of 88.688, well within the range of a high quality model. Validation results suggested that all three models perform equally well in terms of main chain stereochemistry and amino acid environment.

**Fig. 3:**
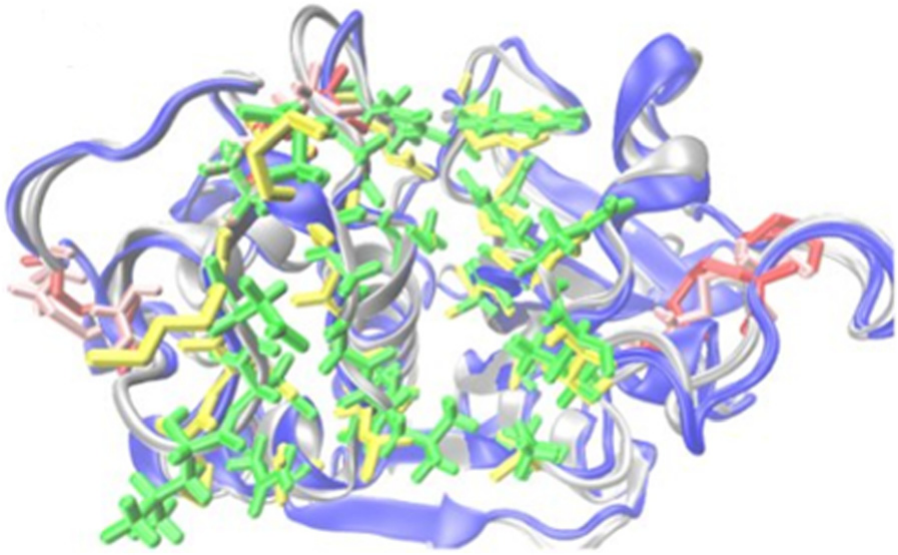
Structural alignment of TsCF1 and 1M6D. Molecule of 1M6D is blue, residues of pocket are yellow, disulfide bonds are red. For TsCF1, molecule is shown in silver, residues of pocket is green, disulfide bonds is pink (the picture was created by VMD1.9.1)

The 3D structure of TsCF1 was very similar to human cathepsin F (1M6D) (Fig. 3). TsCF1 mainly consisted of two domains, R and L domain, with approximately equal size. The R domain consisted primarily of α-helix motifs whereas L domain primarily contained β-barrel motifs. Catalytic active center and substrate binding site of enzyme localized in the junction of the R and L domains, a V-shape cleft. Residues Gln, Cys and His occurred in the central cleft between two domains formed the core of the active site. Besides Gln, Cys and His, there are about 25 residues involved in comprising the binding cleft. Residues located at S1 subsite of the substrate-binding pocket were Gly171, Cys211, Glu212 and Gly213. The S2 subsite was formed by residues Leu215, Pro216, Gly281, Ile307, Asn308, Gly310 and Met359. The S3 subsite was located at Leu207, Asp208, Asp209, Gly214 and Asn218, respectively.

**Fig. 4:**
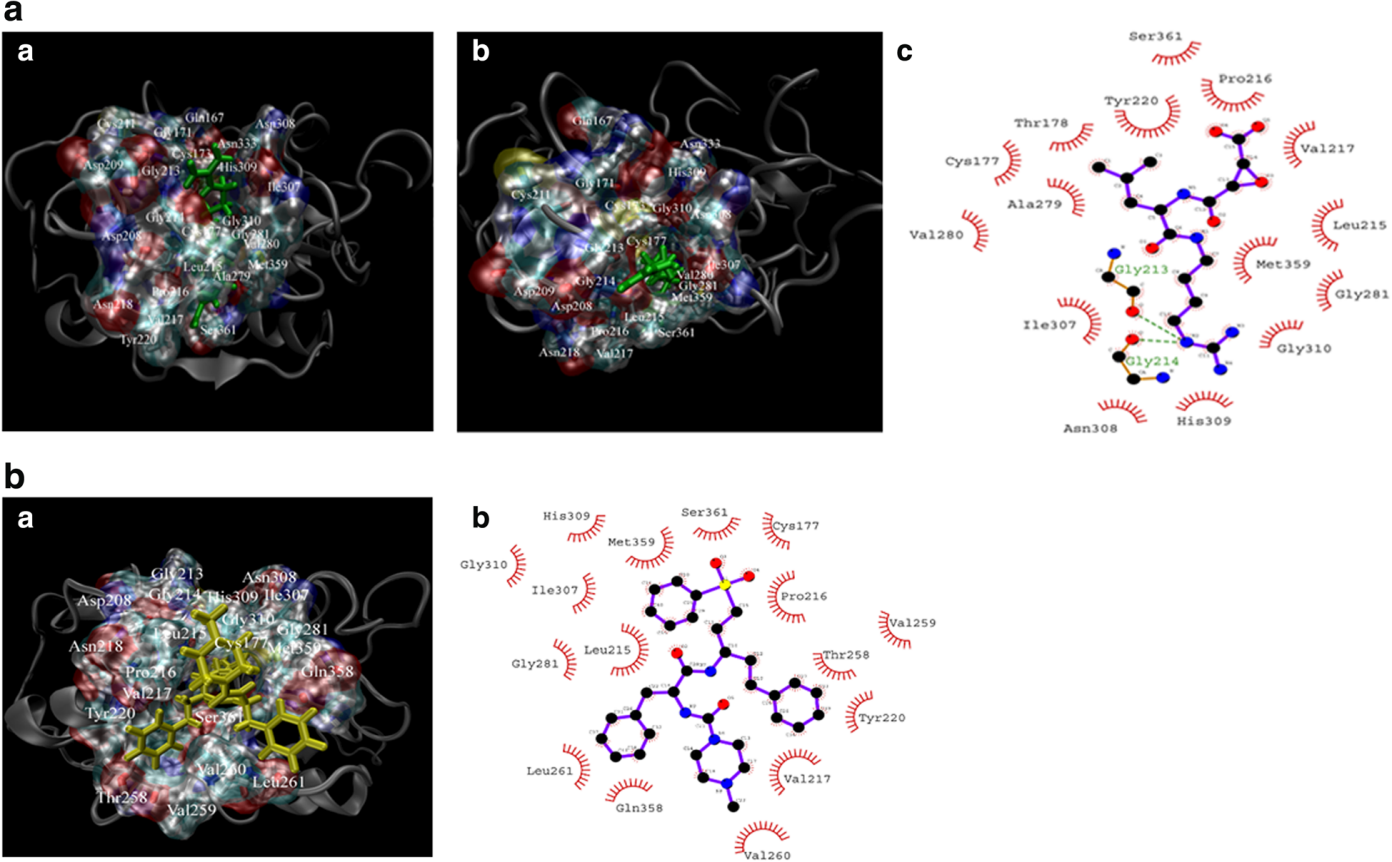
Stereo view of the interactions between TsCF1 (*silver*) and E64 (*green*), K11777 (*yellow*). Surface representation of the pocket of TsCF1, ligand displayed with sticks. **a** Binding mode of E64 and interactions between E64 and TsCF1. *a* and *b* represent the key interactions between TsCF1 and E64, surface representation of modeled TsCF1 active pocket, and inhibitor E64 are shown in *green bonds*. *c* Hydrophobic interactions between TsCF1 and E64. (*a* and *b* was created with VMD1.9.1, and *c* was created with LigPlot v.1.4.5) (color figure online). **b** Binding mode of K11777 and interactions between K11777 and TsCF1. *a* Key interactions between TsCF1 and K11777, surface representation of modeled TsCF1 active pocket, and K11777 are shown in *yellow bonds*. *b* Hydrophobic interactions between TsCF1 and K11777. (*a* was created with VMD1.9.1, and *b* was created with LigPlot v.1.4.5) (color figure online)

In the TsCF1-E64 complex, the S3 subsite consisting of L207, D208, D209 and G214 is in van der Waals contact with E64 In the TsCF1-E64 complex, there are four strong H-bond interactions, the N5…Asn308 amide O, the O3…His309 ND1, the O4…His309 ND1, the N2…Asp209 OD2 (Fig. 4). E64 would insert into pocket and occupies the S2 subsite of TsCF1, then react with residues of S2 by hydrophobic interactions. The binding modes of E64 and K11777 to the respective receptors are schematically displayed in Fig. 4. In the TsCF1-E64 complex, E64 forms H-bond with Gly213 and Gly214, but TsCF1 did not form H-bond with K11777.

### Phylogenetic analysis

**Fig. 5:**
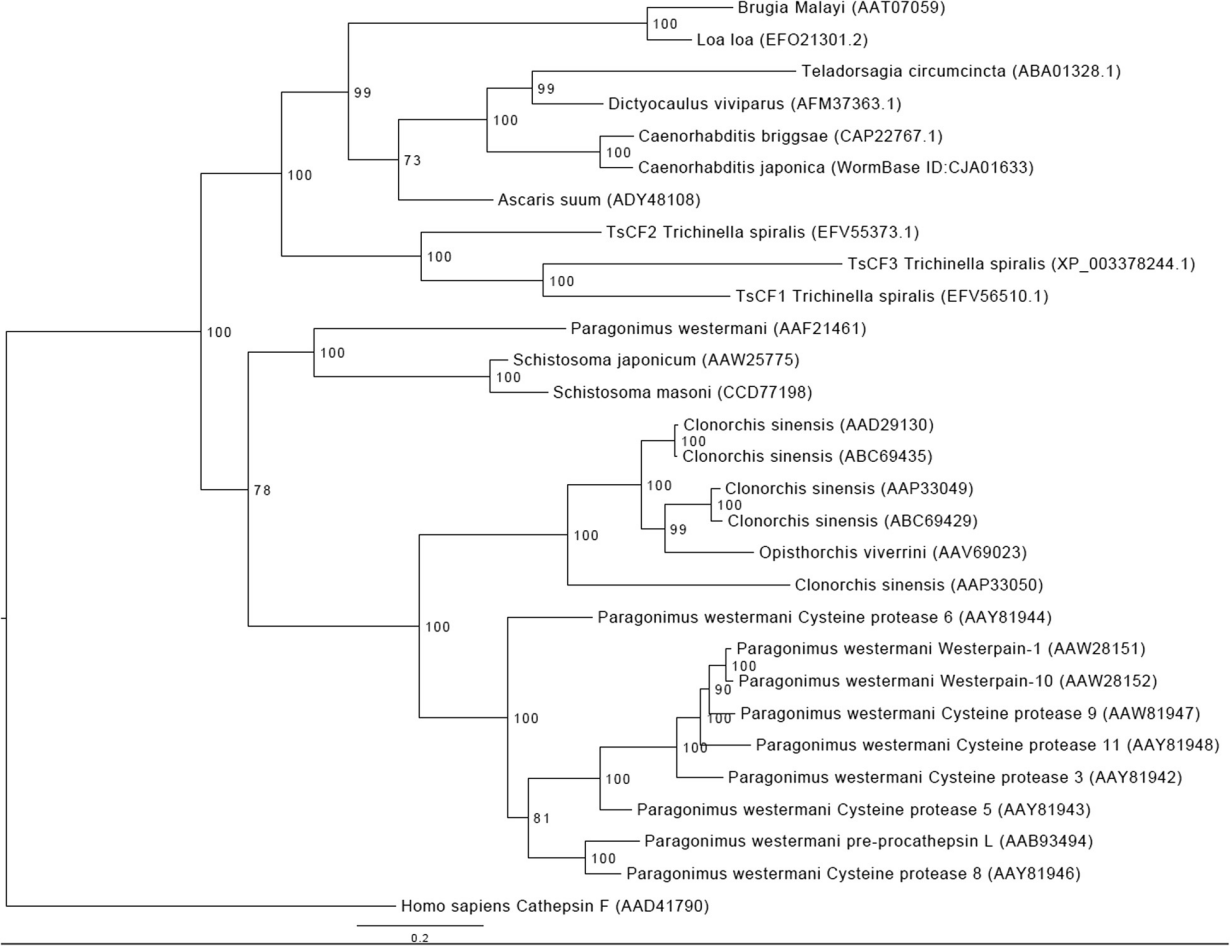
Phylogenetic analysis of cathepsin F family proteins from different species. The GenBank accession number of each cathepsin F is : *T. spiralis* (EFV56510.1), (EFV55373.1), (XP_003378244.1), *Loa loa* (EFO21301.2), *Brugia malayi* (AAT07059), *Teladorsagia circumcincta* (ABA01328), *Dictyocaulus viviparus* (AFM37363.1), *Caenorhabditis briggsae* (CAP22767.1), *Ascaris suum* (ERG86355), *Paragonimus westermani* (AAF21461), (AAY81944), (AAW28151), (AAW28152), (AAW81947), (AAY81948), (AAY81942), (AAY81943), (AAY81946), (AAB93494), *Schistosoma japonicum* (AAW25775), *Schistosoma mansoni* (CCD77198), *Clonorchis sinensis* (AAD29130), (ABC69435), (AAP33049), (ABC69429), (AAP33050), *Opisthorchis viverrini* (AAV69023O) in GenBank and *Caenorhabditis japonica* (Wormbase ID: CJA01633) in wormbase. The tree was rooted using *Homo sapiens* (AAD41790)

TsCF1 showed more than 40 % identity with cathepsin F from other parasitic helminths. Phylogenetic analysis indicated that cathepsin F located in the different clade with nematode cathepsin F. Multiple sequence alignments of cathepsin F clearly demonstrated that residues of catehpsin F were conserved. The conserved motifs, including ERFNAQ, GNFN and GCNGG were screened by alignment of the cathepsin Fs from *H. sapiens* (1M6D), TsCF1, TsCF2 and TsCF3. The active pocket contains the core residues Gln, Cys and His. Phylogenetic analysis of cathepsin F family proteins from different helminth species revealed that two clans existed, one was nematoda clan and the other was trematoda clan (Fig. 5). TsCFs were positioned just at the junction of nematode and trematode.

### Expression of rTsCF1 and western blot analysis

**Fig. 6:**
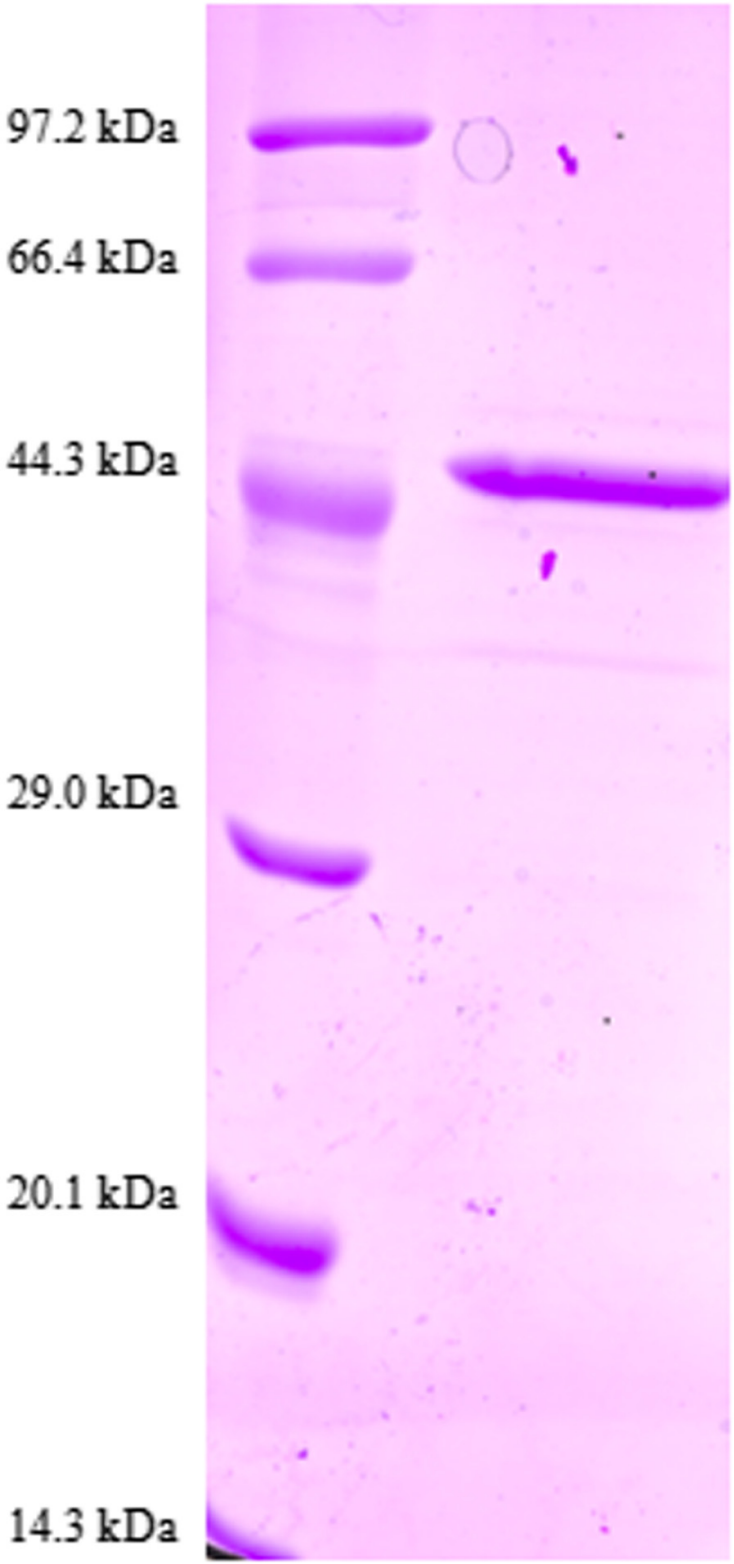
SDS-PAGE analysis of recombinant TsCF1 protein. M: Protein molecular weight marker; 1: purified recombinant protein by Ni-NTA

**Fig. 7:**
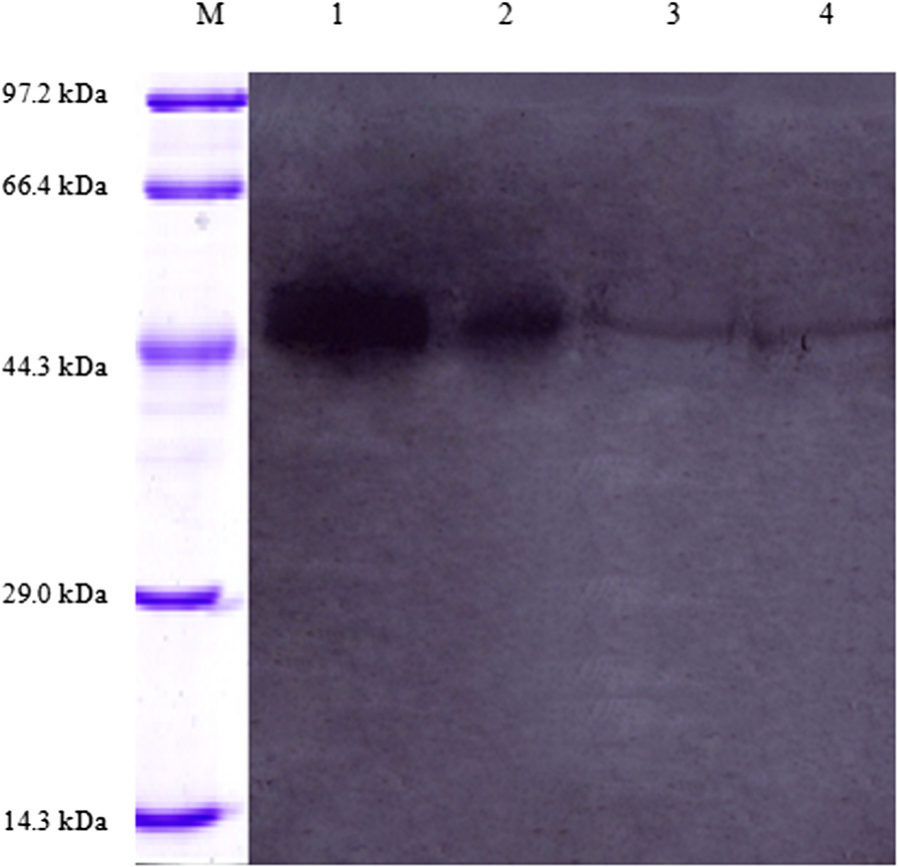
Western blot analysis of antigens of *T. spiralis.* M: Protein molecular weight Marker; 1: rTsCF1; 2: crude extract of *T. spiralis* muscle larvae; 3: ES products of ML; 4: crude extract of *T. spiralis* adult

A cDNA fragment of TsCF1 coding the proregion and C1A mature domain was cloned into pET-30a and transformed into *E. coli* BL21 (DE3). After induction with IPTG, a fusion protein was expressed as inclusion body form. SDS-PAGE analysis of rTsCF1 revealed a 45 kDa band which was consistent with the predicted size (Fig. 6) and MALDI-TOF/TOF analysis indicated nine peptides matched TsCF1. Anti-rTsCF1 rabbit serum reacted with recombinant TsCF1,crude extract of *T. spiralis* adult, muscle larvae and excretory/secretory products of muscle larvae (Fig. 7). And the react band was about 46 kDa, suggesting that this protein existed in different stages of *T. spiralis*.

### Immunolocalization of TsCF1

**Fig. 8:**
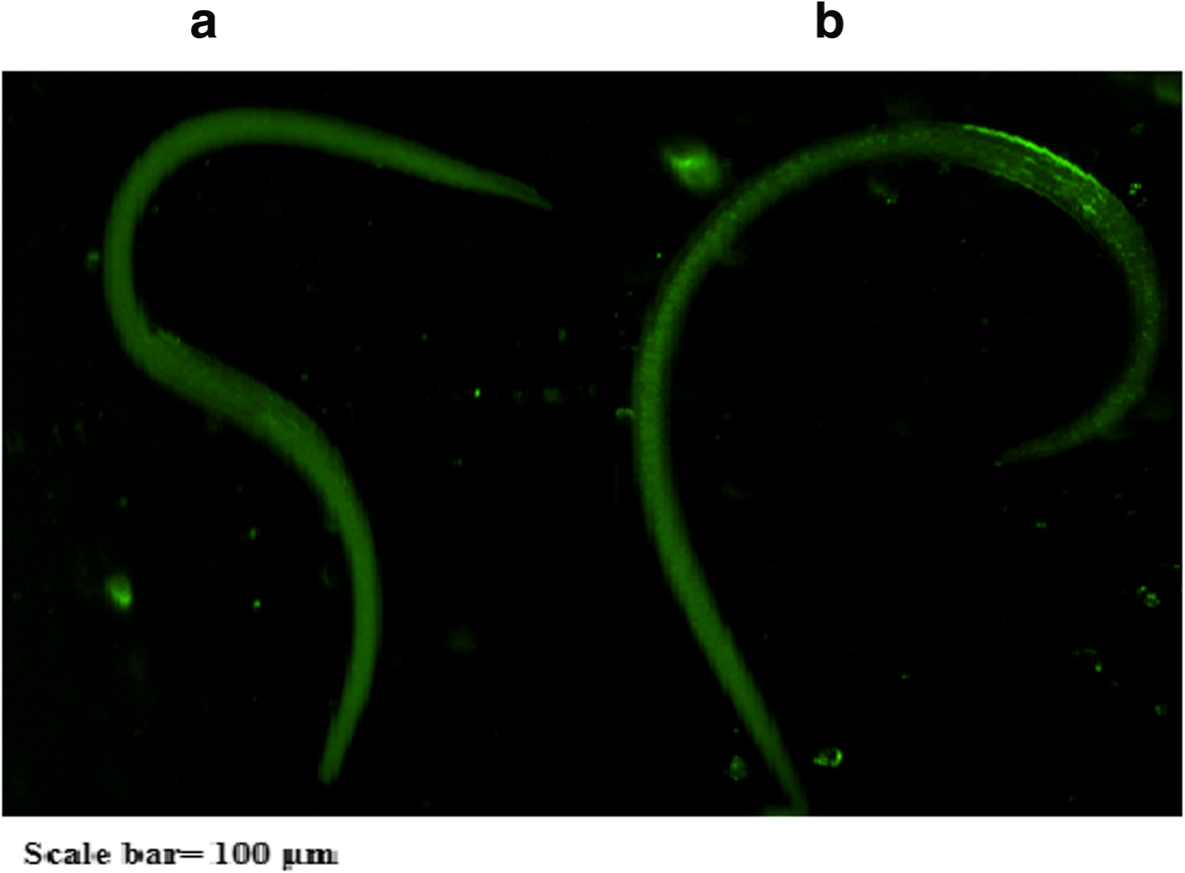
Immunlocalization of TsCF1 in *T. spiralis* muscle larvae. **a** serum of healthy rabbit **b** anti-rTsCF1 serum

The immunolocalization result with the whole parasite showed that the intense green fluorescent staining using anti-rTsCF1 was observed at the cuticle and also slightly at some stichocytes of muscle larvae (Fig. 8).

### Enzymatic characterization of rTsCF1

**Fig. 9:**
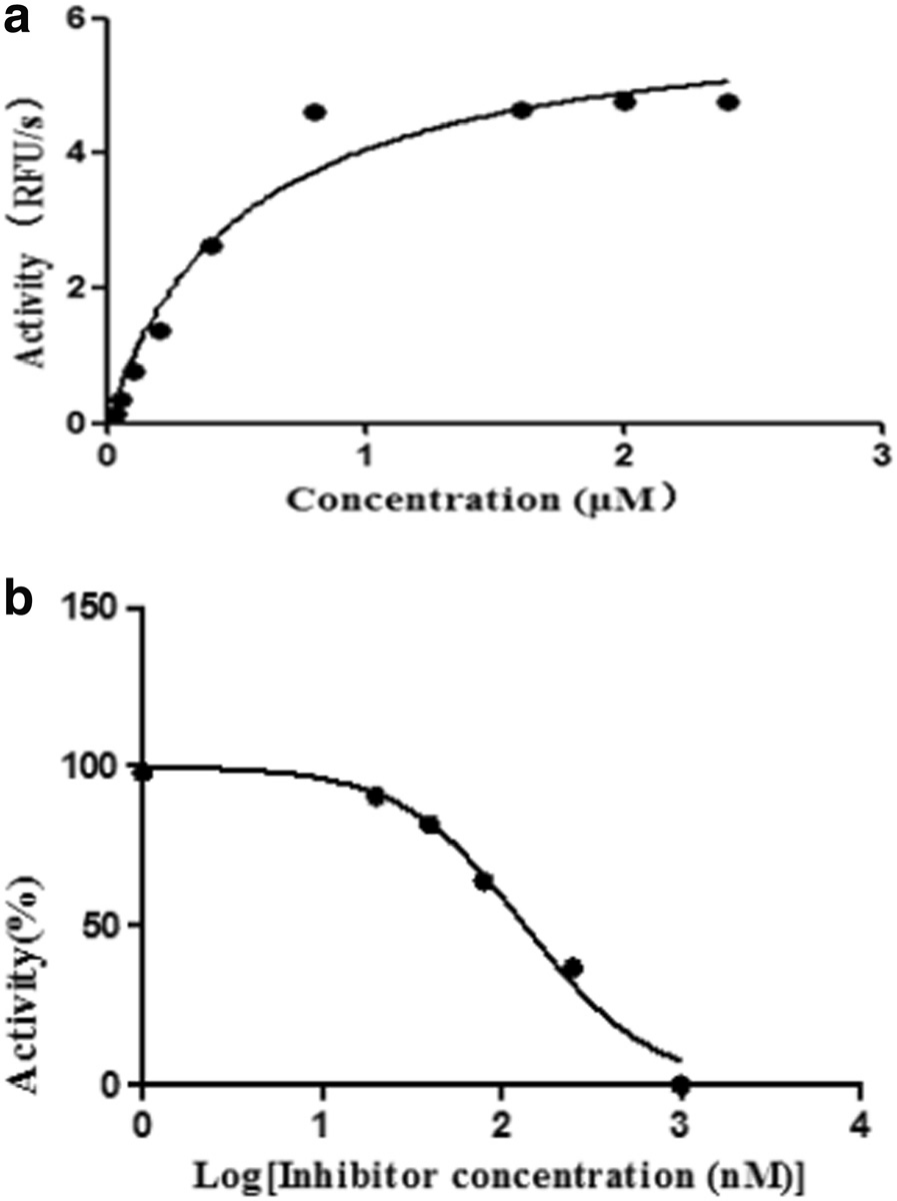
Enzymatic activity of purified rTsCF1. **a** rTsCF1 activity. The concentration of substrate [Z-Phe-Arg-AMC] was plotted on the *x*-axis; the Z-Phe-Arg-AMC in relative fluorescence units (RFU)/s was plotted on the *y*-axis. **b** The curve of inhibitory activity of E64 with rTsCF1. The concentration of E64 was plotted on the *x*-axis; the relative activity of rTsCF1 with E64 is plotted on the *y*-axis

The specific synthetic peptide substrate for cathepsin L like group, Z-Phe-Arg-AMC, was used to investigated the function of the rTsCF1. The peptide was hydrolyzed by rTsCF1 at pH 5.5 with an apparent of Km = 0.5091 μM and Vmax = 6.12 RFU/s μM (Fig. 9a). The peptidase inhibitor E64 which is known to inhibit cysteine proteases of clan CA, family C1, was investigated on the rTsCF1 (Fig.9b). The compound strongly inhibited the enzyme with the IC_50_ value of 135.50 ± 16.90 nM.

## Discussion

Cysteine proteases originated from parasite have a critical role in pathogenesis involved in molting, tissue penetration, nutrition metabolism, immune evasion and immune modulation [29]. It is reported recently that a cysteine protease (ATG4B) of *T. spiralis* could be recognized by early *T. spiralis* infection sera and suggested to be a target for vaccine and immunodiagnostic antigen [30]. *T. spiralis* genome draft was published in 2011 [31] and it is now possible to identify the target gene from genome database. In this study, the *T. spiralis* genome database was mined for searching cathepsin F and three cathepsin F-like proteases were identified. In human, only one cathepsin F was found which is different from other cysteine proteases in that there is a cystatin like domain in the unique very long proregion [32]. This gene was suggested to be a fusion product between an ancestral cathepsin and cystatin gene [33]. The structure of TsCF2 is similar to human cathepsin F, however the cystatin like domain was absent in TsCF1 and TsCF3. Thus the appreciable differences among cathepsin Fs may provide information in drug design for treating *T. spiralis* infection in human.

While the distribution of cathepsin F varies significantly among parasitic helminths, which means that there is a significant diversity among species. As to platyhelminth flukes, the expression pattern of cathepsin F in trematode is variable. In *Schistosoma japonicum* and *Schistosoma mansoni* only one cathepsin F similar to human cathepsin F was identified. *Fasciola hepatica* and *Fasciola gigantica* did not express cathepsin F by database retrieval. However *Opisthorchis viverrini* parasitized in the bile duct of host, expressed one cathepsin F cysteine protease. Intriguingly, the cathepsin F genes in *Clonorchis sinensis* and *Paragonimus westermani* are existed in a pattern of superfamily, which may be correlated with gene expansion events and the function in parasitism. In the nematode, the expression of cathepsin F varies significantly. Three cathepsin F genes were predicted to exist in the genome of *Strongyloides stercoralis.* As to the other filarial nematode *Loa loa,* two cathepsin isoforms existed in the genome; and only one cathepsin F gene was existed in *Ascaris suum*, *Brugia malayi*, *Teladorsagi circumcincta*, *Dictyocaulus viviparus* and *Caenorhabditis elegan*. When the genome database of *Toxocara canis* and *Trichuris trichura* were searched, no cathepsin F was obtained. This may be due to imperfect gene annotation or cathepsin F was authentic in existence. According to the cestode genome data, cathepsin F was not existed within cestode, it may have alternative protease to substitute function of cathepsin F. This phenomenon may be related to the specific anatomical structure. Previously reports demonstrated that cathespin F was associated with nutrition uptake and cathepsin F existed in the gut of parasite. Whereas cestode does not have alimentary canal and oral cavity, which has distinct morphological and histological characterizations thus depending on the body wall for nutrients uptake, and cathepsin B and cathepsin L were abundantly expressed in the tapeworms. In phylogenetic analysis, TsCFs were positioned just at the junction of nematode and trematode. This coincides with the phylogenetic position of *T. spiralis* since *T. spiralis* belongs to the clade I of nematode, located just adjacent to platyhelminth trematode species [34].

The analogous expansion of cathepsin Ls were identified in *F. hepatica* previously, the trematode exerted different functions to survive in the specific tissues of parasitized host. A variety of cathepsin Ls existed in *F. hepatica* as a result of gene duplication and divergent evolution events [35]. Whether the existence of three cathepsin Fs in *T. spiralis* is related to a gene expansion event or not requires futher study.

To date, cathepsin Fs have been functionally characterized in parasitic trematode and nematode [10–13]. Cytochemical studies revealed that cathepsin Fs of trematode localized mainly in the gastrodermis of the gut and participated in extracellular digestion of ingested host tissues and nutrition uptake [11, 12, 36]. Studies also showed that secreted cathepsin F derived from the L4 stage *T. circumcincta* and CsCP derived from *C. sinensis* showed immunogenic characteristic [13, 37]. TsCF1 was localized in the cuticle and stichosome of *T. spiralis* ML and thus might play a pivotal role in interaction between parasite and host.

RT-PCR results indicated TsCF1 was consecutively transcribed during all *T. spiralis* developmental stages including ML, Ad and NBL at expression level. Western blots analysis showed that anti-rTsCF1 serum recognized the native protein in crude antigens of Ad, ML and ES antigens of ML, indicating that TsCF1 is one component of both the crude and ES proteins from *T. spiralis*. These coincide with the data from the global transcriptome analysis of *T. spiralis* [38]. The rTsCF1 was expressed in *E. coli* system as inclusion body form and showed substantial enzyme activity after renaturation. This activity could be inhibited by inhibitor E-64.

Differentiation in the binding modes of E64 to respective enzymes may be due to different pocket structures induced by different residues of each pocket. Because of the similar structure of the Cα backbone chains of pocket, in all complexes, there is a set of similar interactions between ligands and TsCF1 active pocket. According to the previous research, K11777 was a potent specific inhibitor of cathepsin B and preliminary analysis predicted that K11777 could not inhibit TsCF1 activity.

## Conclusions

This is the first work to characterize the cathepsin F-like protease TsCF1 from *T. spiralis*. TsCF1 was expressed in all stages of *T. spiralis*, and localized in the cuticle and stichosome. The recombinant TsCF1 protein demonstrated substantial enzyme activity to Z-Phe-Arg-AMC substrate with pH 5.5 and this activity could be inhibited by cysteine protease inhibitor E-64. These results indicated that TsCF1 might play a role in the life cycle of *T. spiralis* and could be used as a potential vaccine candidate and drug target against *T. spiralis* infection*.* The biological function of the enzyme involved in host-parasite interface in vivo needs to be further studied.
